# (*E*)-2-[1-(1-Benzothio­phen-2-yl)ethyl­idene]-*N*-phenyl­hydrazinecarboxamide

**DOI:** 10.1107/S1600536811037457

**Published:** 2011-09-20

**Authors:** Safa’a Faris Kayed, Yang Farina, Jim Simpson, Ibrahim Baba

**Affiliations:** a School of Chemical Sciences and Food Technology, Faculty of Science and Technology, Universiti Kebangsaan Malaysia, 43600 UKM, Bangi, Selangor, Malaysia; b Department of Chemistry, University of Otago, PO Box 56, Dunedin 9054, New Zealand

## Abstract

The title compound, C_17_H_15_N_3_OS, crystallizes with two unique mol­ecules, denoted 1 and 2, in the asymmetric unit. The two mol­ecules are closely similar and overlay with an r.m.s. deviation of 0.053 Å. Both mol­ecules adopt *E* configurations with respect to the C=N bonds. The dihedral angles between the benzothio­phene groups and N-bound phenyl rings are 36.36 (9)° for mol­ecule 1 and 29.71 (9)° for mol­ecule 2. The C=N—NH—C(O)NH ethyl­idene–hydrazinecarboxamide units are also reasonably planar, with r.m.s. deviations of 0.061 and 0.056 Å, respectively, for the two mol­ecules. The methyl substituents lie 0.338 (3) and 0.396 (3) Å, respectively, from these planes. The C=N—NH—C(O)NH planes are inclined to the phenyl rings at 13.65 (11) and 15.56 (11)°, respectively, in mol­ecules 1 and 2. This conformation is enhanced by weak intra­molecular C—H⋯O hydrogen bonds between *ortho*-H atoms of the two phenyl rings and the carbonyl O atoms, which generate *S*(6) rings in each mol­ecule. In the crystal, pairs of mol­ecules are linked by pairs of inter­molecular N—H⋯O hydrogen bonds into dimers. Alternating dimers are further inter­connected by weak C—H⋯O contacts into zigzag rows along *b*. The rows are stacked along *a* by C—H⋯π contacts involving the benzene ring from molecule 2 and the thiophene ring from molecule 1 of adjacent benzothio­phene units.

## Related literature

For background to the biological activity of semicarbazones, see: Alam *et al.* (2010 ▶); Sharma *et al.* (2006 ▶); Siji *et al.* (2010 ▶); Sriram *et al.* (2004 ▶). For related structures, see: Beraldo *et al.* (2001 ▶); Fun *et al.* (2009*a* ▶,*b* ▶); Mendoza-Meroño *et al.* (2011 ▶). For graph-set analysis of hydrogen bonding, see: Bernstein *et al.* (1995 ▶).
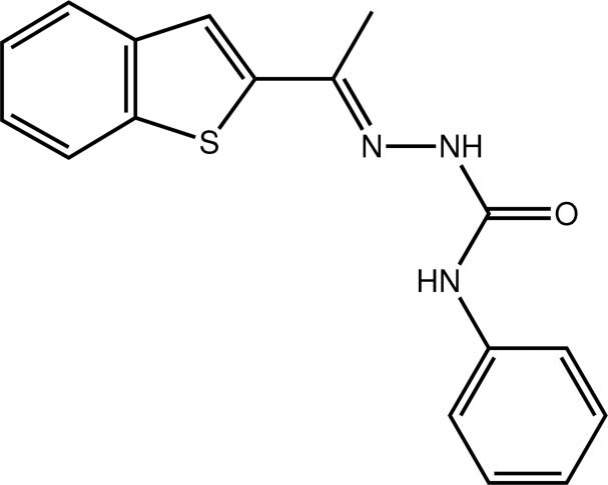


## Experimental

### 

#### Crystal data

C_17_H_15_N_3_OS
*M* *_r_* = 309.38
Triclinic, 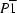

*a* = 9.8858 (3) Å
*b* = 13.2737 (5) Å
*c* = 13.6121 (8) Å
α = 113.961 (3)°
β = 98.153 (3)°
γ = 107.778 (2)°
*V* = 1480.02 (11) Å^3^
*Z* = 4
Mo *K*α radiation
μ = 0.22 mm^−1^
*T* = 89 K
0.38 × 0.14 × 0.05 mm

#### Data collection

Bruker APEXII CCD area-detector diffractometer
Absorption correction: multi-scan (*SADABS*; Bruker, 2009 ▶) *T*_min_ = 0.826, *T*_max_ = 1.000
25263 measured reflections
8650 independent reflections
5747 reflections with *I* > 2σ(*I*)
*R*_int_ = 0.052

#### Refinement

*R*[*F* ^2^ > 2σ(*F* ^2^)] = 0.054
*wR*(*F* ^2^) = 0.147
*S* = 1.07
8650 reflections
411 parameters
H atoms treated by a mixture of independent and constrained refinement
Δρ_max_ = 0.70 e Å^−3^
Δρ_min_ = −0.37 e Å^−3^

### 

Data collection: *APEX2* (Bruker, 2009 ▶); cell refinement: *APEX2* and *SAINT* (Bruker, 2009 ▶); data reduction: *SAINT*; program(s) used to solve structure: *SHELXS97* (Sheldrick, 2008 ▶) and *TITAN2000* (Hunter & Simpson, 1999 ▶); program(s) used to refine structure: *SHELXL97* (Sheldrick, 2008 ▶) and *TITAN2000*; molecular graphics: *SHELXTL* (Sheldrick, 2008 ▶) and *Mercury* (Macrae *et al.*, 2008 ▶); software used to prepare material for publication: *SHELXL97*, *enCIFer* (Allen *et al.*, 2004 ▶), *PLATON* (Spek, 2009 ▶) and *publCIF* (Westrip, 2010 ▶).

## Supplementary Material

Crystal structure: contains datablock(s) global, I. DOI: 10.1107/S1600536811037457/tk2786sup1.cif

Structure factors: contains datablock(s) I. DOI: 10.1107/S1600536811037457/tk2786Isup2.hkl

Supplementary material file. DOI: 10.1107/S1600536811037457/tk2786Isup3.cml

Additional supplementary materials:  crystallographic information; 3D view; checkCIF report

## Figures and Tables

**Table 1:** Hydrogen-bond geometry (Å, °) *Cg*1 is the centroid of the S11, C11, C12, C13, C18 ring.

*D*—H⋯*A*	*D*—H	H⋯*A*	*D*⋯*A*	*D*—H⋯*A*
N12—H12*N*⋯O21	0.97 (2)	1.91 (2)	2.847 (2)	162.1 (19)
N22—H22*N*⋯O11	0.89 (2)	1.99 (2)	2.840 (2)	158 (2)
C113—H113⋯O11	0.95	2.29	2.886 (2)	120
C213—H213⋯O21	0.95	2.26	2.871 (2)	121
C15—H15⋯O11^i^	0.95	2.62	3.435 (2)	144
C24—H24⋯*Cg*1^ii^	0.95	2.80	3.482 (2)	130

Symmetry codes: (i) 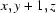; (ii) 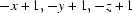.

**Fig. 1.**
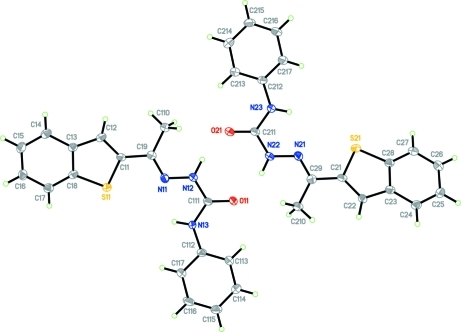
The asymmetric unit of the title compound with ellipsoids drawn at the 50% probability level.

**Fig. 2.**
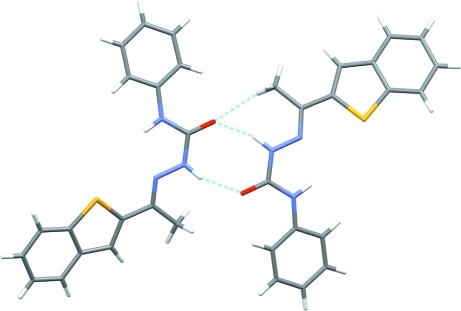
Dimers formed by the two unique molecules.Hydrogen bonds are shown as dashed lines.

**Fig. 3.**
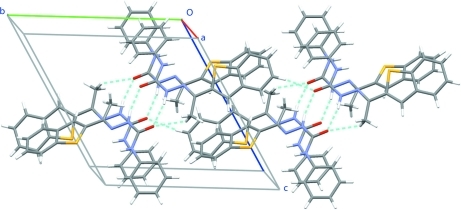
Crystal packing with hydrogen bonds drawn as dashed lines.

## supplementary crystallographic information

### Comment

Semicarbazones are of considerable interest because of their wide spectrum of biological applications, and display anticonvulsant (Alam *et al.*, 2010), antitubercular (Sriram *et al.*, 2004) and antimicrobial activity (Siji *et al.*, 2010). The biological activity of semicarbazones is considered to be due to their ability to form chelate complexes with transition metals (Sharma *et al.*, 2006).In view of the importance of these compounds, we report here the structure of the title semicarbazone derivative (Fig. 1).

The asymmetric unit of the title compound contains two molecules, 1 and 2. The benzothiophene group and the C9=N1—N2—C11(O1)N3 semicarbazone units are almost coplanar with N1—C9—C1—S1 torsion angles of -13.0 (2)° for 1 and -9.3 (2)° for 2. The phenyl rings show somewhat greater coplanarity with the semicarbazone linking units the C11—N3—C12—C13 angles being -13.3 (3)° for 1 and 5.4 (3)° for 2. The dihedral angles between the benzothiophene groups and the phenyl rings are 36.36 (7)° for 1 and 29.71 (8)° for 2. Both molecules adopt *E* configurations with respect to the C=N bonds. There are no important differences in the bond lengths and angles between the two unique molecules which overlay with an r.m.s. deviation of 0.053 Å (Macrae *et al.*, 2008). The bond distances and angles are in a good agreement with values reported for similar structures (Beraldo *et al.*, 2001; Fun *et al.*, 2009*a*,*b*; Mendoza-Meroño *et al.* 2011). Intramolecular C113–H113···O11 and C113–H113···O11 hydrogen bonds are observed in both molecules and generate S(6) rings (Bernstein *et al.* 1995).

In the crystal structure, intermolecular N12—H12N···O21 and N22—H22N···O11 hydrogen bonds (Table 1) link the molecules into dimers (Fig. 2). A weak C110—H11A..O21 interaction further strengthens the dimer unit for molecule 1. Alternating dimers are further interconnected by weak C15–H15···O11 contacts generating zigzag rows along *b*. C24—H24..π contacts to the S11, C11, C12, C13, C18 thiophene rings of adjacent molecules form stacks along *a*, (Fig. 3).

### Experimental

4-Phenyl-3-semicarbazide (1.51 g, 10 mmol) was dissolved in boiling ethanol (40 ml). A solution of 2-acetylbenzothiophene (1.77 g, 10 mmol) in ethanol (30 ml) was added to the 4-phenylsemicarbazide solution followed by the addition of three drops of sulphuric acid. The mixture was heated under reflux with stirring for 2 h. The solid product which separated upon cooling was filtered and recrystallized from a 1:1 mixture of dimethylsulphoxide and ethanol.

### Refinement

H atoms bound to N1 and N3 were located in an electron density map and their coordinates were refined freely with *U*_iso_= 1.2*U*_eq_ (N). All H-atoms bound to carbon were refined using a riding model with d(C—H) = 0.95 Å, *U*_iso_=1.2*U*_eq_ (C) for aromatic and 0.98 Å, *U*_iso_ = 1.5*U*_eq_ (C) for the CH_3_ H atoms.

### Crystal data

**Table:**

C_17_H_15_N_3_OS	*Z* = 4
*M**_r_* = 309.38	*F*(000) = 648
Triclinic, *P*1	*D*_x_ = 1.388 Mg m^−^^3^
Hall symbol: -P 1	Mo *K*α radiation, λ = 0.71073 Å
*a* = 9.8858 (3) Å	Cell parameters from 3121 reflections
*b* = 13.2737 (5) Å	θ = 2.3–24.7°
*c* = 13.6121 (8) Å	µ = 0.22 mm^−^^1^
α = 113.961 (3)°	*T* = 89 K
β = 98.153 (3)°	Rectangular plate, colourless
γ = 107.778 (2)°	0.38 × 0.14 × 0.05 mm
*V* = 1480.02 (11) Å^3^	

### Data collection

**Table:**

Bruker APEXII CCD area-detector diffractometer	8650 independent reflections
Radiation source: fine-focus sealed tube	5747 reflections with *I* > 2σ(*I*)
graphite	*R*_int_ = 0.052
ω scans	θ_max_ = 30.1°, θ_min_ = 2.3°
Absorption correction: multi-scan (*SADABS*; Bruker, 2009)	*h* = −13→13
*T*_min_ = 0.826, *T*_max_ = 1.000	*k* = −18→18
25263 measured reflections	*l* = −19→16

### Refinement

**Table:**

Refinement on *F*^2^	Primary atom site location: structure-invariant direct methods
Least-squares matrix: full	Secondary atom site location: difference Fourier map
*R*[*F*^2^ > 2σ(*F*^2^)] = 0.054	Hydrogen site location: inferred from neighbouring sites
*wR*(*F*^2^) = 0.147	H atoms treated by a mixture of independent and constrained refinement
*S* = 1.07	*w* = 1/[σ^2^(*F*_o_^2^) + (0.0651*P*)^2^ + 0.0525*P*] where *P* = (*F*_o_^2^ + 2*F*_c_^2^)/3
8650 reflections	(Δ/σ)_max_ < 0.001
411 parameters	Δρ_max_ = 0.70 e Å^−^^3^
0 restraints	Δρ_min_ = −0.37 e Å^−^^3^

### Special details

**Table:**

Geometry. All e.s.d.'s (except the e.s.d. in the dihedral angle between two l.s. planes) are estimated using the full covariance matrix. The cell e.s.d.'s are taken into account individually in the estimation of e.s.d.'s in distances, angles and torsion angles; correlations between e.s.d.'s in cell parameters are only used when they are defined by crystal symmetry. An approximate (isotropic) treatment of cell e.s.d.'s is used for estimating e.s.d.'s involving l.s. planes.
Refinement. Refinement of *F*^2^ against ALL reflections. The weighted *R*-factor *wR* and goodness of fit *S* are based on *F*^2^, conventional *R*-factors *R* are based on *F*, with *F* set to zero for negative *F*^2^. The threshold expression of *F*^2^ > σ(*F*^2^) is used only for calculating *R*-factors(gt) *etc*. and is not relevant to the choice of reflections for refinement. *R*-factors based on *F*^2^ are statistically about twice as large as those based on *F*, and *R*- factors based on ALL data will be even larger.

### Fractional atomic coordinates and isotropic or equivalent isotropic displacement parameters (Å^2^)

**Table:**

	*x*	*y*	*z*	*U*_iso_*/*U*_eq_	
S11	0.31292 (6)	1.02673 (4)	0.78076 (4)	0.02141 (13)	
C11	0.2261 (2)	0.93460 (17)	0.63600 (16)	0.0182 (4)	
C12	0.1846 (2)	0.99340 (18)	0.58266 (17)	0.0199 (4)	
H12	0.1351	0.9563	0.5041	0.024*	
C13	0.2253 (2)	1.11904 (17)	0.66052 (16)	0.0180 (4)	
C14	0.2057 (2)	1.20934 (18)	0.63901 (18)	0.0223 (4)	
H14	0.1568	1.1906	0.5647	0.027*	
C15	0.2576 (2)	1.32550 (19)	0.72612 (18)	0.0251 (5)	
H15	0.2451	1.3866	0.7112	0.030*	
C16	0.3287 (2)	1.35423 (18)	0.83629 (18)	0.0240 (4)	
H16	0.3640	1.4347	0.8951	0.029*	
C17	0.3481 (2)	1.26718 (18)	0.86079 (17)	0.0221 (4)	
H17	0.3952	1.2866	0.9358	0.027*	
C18	0.2966 (2)	1.14948 (18)	0.77204 (17)	0.0201 (4)	
C19	0.2066 (2)	0.80832 (17)	0.58560 (16)	0.0181 (4)	
C110	0.1043 (2)	0.72127 (18)	0.46823 (16)	0.0237 (4)	
H11A	0.1562	0.6766	0.4240	0.035*	
H11B	0.0740	0.7653	0.4328	0.035*	
H11C	0.0157	0.6649	0.4710	0.035*	
N11	0.28160 (17)	0.78150 (14)	0.64940 (13)	0.0190 (3)	
N12	0.26205 (18)	0.66309 (15)	0.60819 (14)	0.0200 (4)	
H12N	0.193 (2)	0.6052 (19)	0.5336 (18)	0.024*	
C111	0.3580 (2)	0.63400 (17)	0.66381 (16)	0.0189 (4)	
O11	0.34514 (15)	0.52858 (12)	0.62557 (11)	0.0231 (3)	
N13	0.46505 (19)	0.72820 (15)	0.75850 (14)	0.0212 (4)	
H13N	0.460 (2)	0.798 (2)	0.7734 (18)	0.025*	
C112	0.5902 (2)	0.72786 (18)	0.82322 (16)	0.0200 (4)	
C113	0.6083 (2)	0.62516 (19)	0.81270 (19)	0.0298 (5)	
H113	0.5327	0.5479	0.7611	0.036*	
C114	0.7391 (3)	0.6363 (2)	0.8788 (2)	0.0356 (6)	
H114	0.7524	0.5656	0.8703	0.043*	
C115	0.8490 (2)	0.74708 (19)	0.95598 (17)	0.0246 (4)	
H115	0.9372	0.7532	1.0007	0.030*	
C116	0.8291 (2)	0.84825 (19)	0.96713 (17)	0.0277 (5)	
H116	0.9038	0.9253	1.0205	0.033*	
C117	0.7008 (2)	0.83968 (19)	0.90140 (18)	0.0300 (5)	
H117	0.6887	0.9108	0.9100	0.036*	
S21	0.10557 (5)	−0.03449 (5)	0.26492 (4)	0.02144 (13)	
C21	0.2802 (2)	0.07844 (17)	0.35461 (15)	0.0183 (4)	
C22	0.3828 (2)	0.03329 (17)	0.37642 (16)	0.0172 (4)	
H22	0.4829	0.0820	0.4238	0.021*	
C23	0.3203 (2)	−0.09632 (18)	0.31879 (16)	0.0195 (4)	
C24	0.3903 (2)	−0.17387 (18)	0.31967 (17)	0.0229 (4)	
H24	0.4920	−0.1417	0.3625	0.027*	
C25	0.3106 (2)	−0.29689 (19)	0.25799 (17)	0.0248 (4)	
H25	0.3577	−0.3494	0.2583	0.030*	
C26	0.1598 (2)	−0.34491 (19)	0.19454 (17)	0.0247 (4)	
H26	0.1062	−0.4297	0.1523	0.030*	
C27	0.0886 (2)	−0.27078 (18)	0.19275 (17)	0.0233 (4)	
H27	−0.0134	−0.3038	0.1502	0.028*	
C28	0.1692 (2)	−0.14626 (18)	0.25469 (16)	0.0193 (4)	
C29	0.3002 (2)	0.20465 (18)	0.40077 (16)	0.0191 (4)	
C210	0.4392 (2)	0.30265 (18)	0.49234 (16)	0.0228 (4)	
H21A	0.4728	0.3703	0.4764	0.034*	
H21B	0.5173	0.2720	0.4956	0.034*	
H21C	0.4186	0.3298	0.5649	0.034*	
N21	0.18945 (18)	0.22163 (14)	0.35750 (13)	0.0202 (4)	
N22	0.19800 (19)	0.33747 (15)	0.40139 (14)	0.0225 (4)	
H22N	0.261 (2)	0.388 (2)	0.4711 (19)	0.027*	
C211	0.0935 (2)	0.36293 (18)	0.34891 (16)	0.0207 (4)	
O21	0.09507 (17)	0.46410 (13)	0.38988 (12)	0.0291 (4)	
N23	−0.00679 (18)	0.26752 (15)	0.25064 (14)	0.0203 (4)	
H23N	0.005 (2)	0.201 (2)	0.2342 (18)	0.024*	
C212	−0.1117 (2)	0.26998 (18)	0.17048 (16)	0.0198 (4)	
C213	−0.1193 (2)	0.37503 (19)	0.17685 (17)	0.0238 (4)	
H213	−0.0578	0.4508	0.2406	0.029*	
C214	−0.2185 (2)	0.3675 (2)	0.08827 (18)	0.0287 (5)	
H214	−0.2228	0.4393	0.0915	0.034*	
C215	−0.3112 (2)	0.25807 (19)	−0.00446 (17)	0.0254 (5)	
H215	−0.3781	0.2544	−0.0645	0.030*	
C216	−0.3048 (2)	0.15487 (19)	−0.00818 (18)	0.0297 (5)	
H216	−0.3685	0.0791	−0.0711	0.036*	
C217	−0.2063 (2)	0.15971 (19)	0.07875 (17)	0.0270 (5)	
H217	−0.2038	0.0875	0.0755	0.032*	

### Atomic displacement parameters (Å^2^)

**Table:**

	*U*^11^	*U*^22^	*U*^33^	*U*^12^	*U*^13^	*U*^23^
S11	0.0247 (3)	0.0198 (3)	0.0174 (3)	0.0095 (2)	0.0014 (2)	0.0079 (2)
C11	0.0149 (9)	0.0197 (10)	0.0162 (10)	0.0056 (8)	0.0016 (7)	0.0072 (8)
C12	0.0156 (9)	0.0264 (11)	0.0246 (11)	0.0092 (8)	0.0068 (8)	0.0175 (9)
C13	0.0146 (9)	0.0204 (10)	0.0204 (10)	0.0076 (8)	0.0056 (7)	0.0106 (8)
C14	0.0181 (9)	0.0284 (11)	0.0273 (11)	0.0108 (9)	0.0081 (8)	0.0181 (10)
C15	0.0233 (10)	0.0234 (11)	0.0344 (12)	0.0106 (9)	0.0091 (9)	0.0179 (10)
C16	0.0242 (10)	0.0192 (10)	0.0279 (11)	0.0118 (9)	0.0076 (9)	0.0085 (9)
C17	0.0208 (10)	0.0242 (11)	0.0217 (10)	0.0107 (9)	0.0064 (8)	0.0101 (9)
C18	0.0179 (9)	0.0236 (10)	0.0217 (10)	0.0097 (8)	0.0053 (8)	0.0126 (9)
C19	0.0161 (9)	0.0199 (10)	0.0166 (9)	0.0067 (8)	0.0057 (7)	0.0072 (8)
C110	0.0255 (10)	0.0209 (10)	0.0196 (10)	0.0084 (9)	0.0013 (8)	0.0077 (9)
N11	0.0190 (8)	0.0154 (8)	0.0192 (8)	0.0058 (7)	0.0046 (7)	0.0065 (7)
N12	0.0219 (9)	0.0159 (8)	0.0167 (8)	0.0064 (7)	0.0006 (7)	0.0054 (7)
C111	0.0181 (9)	0.0190 (10)	0.0142 (9)	0.0046 (8)	0.0008 (7)	0.0066 (8)
O11	0.0287 (8)	0.0139 (7)	0.0172 (7)	0.0068 (6)	−0.0029 (6)	0.0035 (6)
N13	0.0250 (9)	0.0142 (8)	0.0180 (9)	0.0091 (7)	−0.0022 (7)	0.0039 (7)
C112	0.0215 (10)	0.0221 (10)	0.0132 (9)	0.0091 (8)	0.0020 (8)	0.0063 (8)
C113	0.0295 (12)	0.0174 (10)	0.0288 (12)	0.0068 (9)	−0.0057 (9)	0.0055 (9)
C114	0.0383 (13)	0.0270 (12)	0.0319 (13)	0.0187 (11)	−0.0051 (10)	0.0064 (10)
C115	0.0231 (10)	0.0307 (12)	0.0180 (10)	0.0124 (9)	0.0029 (8)	0.0097 (9)
C116	0.0242 (11)	0.0255 (11)	0.0193 (11)	0.0023 (9)	−0.0041 (8)	0.0070 (9)
C117	0.0320 (12)	0.0183 (11)	0.0265 (12)	0.0069 (9)	−0.0064 (9)	0.0061 (9)
S21	0.0167 (2)	0.0221 (3)	0.0208 (3)	0.0069 (2)	0.00048 (19)	0.0083 (2)
C21	0.0158 (9)	0.0215 (10)	0.0134 (9)	0.0058 (8)	0.0021 (7)	0.0066 (8)
C22	0.0146 (9)	0.0168 (9)	0.0165 (9)	0.0046 (7)	0.0052 (7)	0.0057 (8)
C23	0.0162 (9)	0.0224 (10)	0.0168 (10)	0.0068 (8)	0.0037 (7)	0.0076 (8)
C24	0.0168 (9)	0.0274 (11)	0.0228 (11)	0.0101 (9)	0.0051 (8)	0.0099 (9)
C25	0.0256 (11)	0.0264 (11)	0.0251 (11)	0.0152 (9)	0.0074 (9)	0.0113 (9)
C26	0.0244 (11)	0.0212 (11)	0.0223 (11)	0.0063 (9)	0.0053 (8)	0.0075 (9)
C27	0.0216 (10)	0.0245 (11)	0.0188 (10)	0.0067 (9)	0.0019 (8)	0.0090 (9)
C28	0.0192 (9)	0.0231 (10)	0.0154 (9)	0.0088 (8)	0.0046 (8)	0.0088 (8)
C29	0.0190 (9)	0.0234 (10)	0.0173 (10)	0.0095 (8)	0.0078 (8)	0.0104 (8)
C210	0.0207 (10)	0.0242 (11)	0.0179 (10)	0.0072 (9)	0.0030 (8)	0.0075 (9)
N21	0.0248 (9)	0.0180 (8)	0.0154 (8)	0.0088 (7)	0.0044 (7)	0.0061 (7)
N22	0.0244 (9)	0.0187 (9)	0.0146 (8)	0.0083 (7)	−0.0023 (7)	0.0019 (7)
C211	0.0213 (10)	0.0197 (10)	0.0152 (10)	0.0059 (8)	0.0009 (8)	0.0062 (8)
O21	0.0336 (9)	0.0218 (8)	0.0188 (8)	0.0127 (7)	−0.0054 (6)	0.0009 (6)
N23	0.0224 (8)	0.0167 (8)	0.0165 (9)	0.0082 (7)	−0.0003 (7)	0.0049 (7)
C212	0.0189 (9)	0.0213 (10)	0.0160 (10)	0.0064 (8)	0.0021 (8)	0.0081 (8)
C213	0.0245 (10)	0.0223 (11)	0.0197 (10)	0.0087 (9)	0.0012 (8)	0.0079 (9)
C214	0.0308 (12)	0.0301 (12)	0.0303 (12)	0.0173 (10)	0.0054 (10)	0.0166 (10)
C215	0.0237 (10)	0.0325 (12)	0.0194 (10)	0.0127 (9)	0.0021 (8)	0.0123 (9)
C216	0.0280 (11)	0.0238 (12)	0.0215 (11)	0.0049 (9)	−0.0059 (9)	0.0050 (9)
C217	0.0295 (11)	0.0203 (11)	0.0220 (11)	0.0068 (9)	−0.0027 (9)	0.0077 (9)

### Geometric parameters (Å, °)

**Table:**

S11—C18	1.734 (2)	S21—C28	1.745 (2)
S11—C11	1.7427 (19)	S21—C21	1.7473 (19)
C11—C12	1.369 (2)	C21—C22	1.381 (2)
C11—C19	1.466 (3)	C21—C29	1.466 (3)
C12—C13	1.449 (3)	C22—C23	1.440 (3)
C12—H12	0.9500	C22—H22	0.9500
C13—C14	1.404 (3)	C23—C24	1.407 (3)
C13—C18	1.410 (3)	C23—C28	1.410 (3)
C14—C15	1.380 (3)	C24—C25	1.380 (3)
C14—H14	0.9500	C24—H24	0.9500
C15—C16	1.400 (3)	C25—C26	1.408 (3)
C15—H15	0.9500	C25—H25	0.9500
C16—C17	1.383 (3)	C26—C27	1.379 (3)
C16—H16	0.9500	C26—H26	0.9500
C17—C18	1.402 (3)	C27—C28	1.397 (3)
C17—H17	0.9500	C27—H27	0.9500
C19—N11	1.289 (2)	C29—N21	1.293 (2)
C19—C110	1.496 (3)	C29—C210	1.497 (3)
C110—H11A	0.9800	C210—H21A	0.9800
C110—H11B	0.9800	C210—H21B	0.9800
C110—H11C	0.9800	C210—H21C	0.9800
N11—N12	1.375 (2)	N21—N22	1.374 (2)
N12—C111	1.371 (2)	N22—C211	1.376 (2)
N12—H12N	0.97 (2)	N22—H22N	0.89 (2)
C111—O11	1.235 (2)	C211—O21	1.221 (2)
C111—N13	1.357 (2)	C211—N23	1.363 (2)
N13—C112	1.416 (2)	N23—C212	1.413 (2)
N13—H13N	0.89 (2)	N23—H23N	0.87 (2)
C112—C113	1.382 (3)	C212—C217	1.387 (3)
C112—C117	1.388 (3)	C212—C213	1.388 (3)
C113—C114	1.396 (3)	C213—C214	1.390 (3)
C113—H113	0.9500	C213—H213	0.9500
C114—C115	1.376 (3)	C214—C215	1.383 (3)
C114—H114	0.9500	C214—H214	0.9500
C115—C116	1.368 (3)	C215—C216	1.372 (3)
C115—H115	0.9500	C215—H215	0.9500
C116—C117	1.389 (3)	C216—C217	1.387 (3)
C116—H116	0.9500	C216—H216	0.9500
C117—H117	0.9500	C217—H217	0.9500
			
C18—S11—C11	91.28 (9)	C28—S21—C21	91.28 (9)
C12—C11—C19	127.84 (18)	C22—C21—C29	128.03 (17)
C12—C11—S11	113.22 (15)	C22—C21—S21	112.81 (14)
C19—C11—S11	118.94 (14)	C29—C21—S21	119.10 (14)
C11—C12—C13	111.86 (18)	C21—C22—C23	112.03 (17)
C11—C12—H12	124.1	C21—C22—H22	124.0
C13—C12—H12	124.1	C23—C22—H22	124.0
C14—C13—C18	118.69 (18)	C24—C23—C28	119.07 (18)
C14—C13—C12	129.36 (18)	C24—C23—C22	128.48 (18)
C18—C13—C12	111.93 (17)	C28—C23—C22	112.45 (17)
C15—C14—C13	119.85 (19)	C25—C24—C23	119.75 (18)
C15—C14—H14	120.1	C25—C24—H24	120.1
C13—C14—H14	120.1	C23—C24—H24	120.1
C14—C15—C16	120.69 (19)	C24—C25—C26	120.27 (19)
C14—C15—H15	119.7	C24—C25—H25	119.9
C16—C15—H15	119.7	C26—C25—H25	119.9
C17—C16—C15	121.00 (19)	C27—C26—C25	121.06 (19)
C17—C16—H16	119.5	C27—C26—H26	119.5
C15—C16—H16	119.5	C25—C26—H26	119.5
C16—C17—C18	118.25 (19)	C26—C27—C28	118.75 (18)
C16—C17—H17	120.9	C26—C27—H27	120.6
C18—C17—H17	120.9	C28—C27—H27	120.6
C17—C18—C13	121.51 (18)	C27—C28—C23	121.10 (18)
C17—C18—S11	126.77 (15)	C27—C28—S21	127.46 (15)
C13—C18—S11	111.71 (15)	C23—C28—S21	111.44 (15)
N11—C19—C11	115.21 (17)	N21—C29—C21	114.78 (17)
N11—C19—C110	124.97 (18)	N21—C29—C210	124.63 (18)
C11—C19—C110	119.82 (16)	C21—C29—C210	120.58 (17)
C19—C110—H11A	109.5	C29—C210—H21A	109.5
C19—C110—H11B	109.5	C29—C210—H21B	109.5
H11A—C110—H11B	109.5	H21A—C210—H21B	109.5
C19—C110—H11C	109.5	C29—C210—H21C	109.5
H11A—C110—H11C	109.5	H21A—C210—H21C	109.5
H11B—C110—H11C	109.5	H21B—C210—H21C	109.5
C19—N11—N12	117.57 (16)	C29—N21—N22	117.60 (16)
C111—N12—N11	119.32 (16)	N21—N22—C211	119.89 (16)
C111—N12—H12N	121.2 (13)	N21—N22—H22N	115.3 (14)
N11—N12—H12N	118.4 (13)	C211—N22—H22N	123.5 (14)
O11—C111—N13	124.78 (17)	O21—C211—N23	124.92 (18)
O11—C111—N12	120.06 (17)	O21—C211—N22	120.69 (18)
N13—C111—N12	115.14 (17)	N23—C211—N22	114.39 (18)
C111—N13—C112	127.34 (17)	C211—N23—C212	127.10 (17)
C111—N13—H13N	113.6 (14)	C211—N23—H23N	113.9 (14)
C112—N13—H13N	118.4 (14)	C212—N23—H23N	118.6 (14)
C113—C112—C117	119.18 (18)	C217—C212—C213	119.81 (18)
C113—C112—N13	124.47 (18)	C217—C212—N23	116.38 (18)
C117—C112—N13	116.35 (18)	C213—C212—N23	123.75 (18)
C112—C113—C114	119.2 (2)	C212—C213—C214	118.85 (19)
C112—C113—H113	120.4	C212—C213—H213	120.6
C114—C113—H113	120.4	C214—C213—H213	120.6
C115—C114—C113	121.6 (2)	C215—C214—C213	121.6 (2)
C115—C114—H114	119.2	C215—C214—H214	119.2
C113—C114—H114	119.2	C213—C214—H214	119.2
C116—C115—C114	118.76 (19)	C216—C215—C214	118.77 (19)
C116—C115—H115	120.6	C216—C215—H215	120.6
C114—C115—H115	120.6	C214—C215—H215	120.6
C115—C116—C117	120.8 (2)	C215—C216—C217	120.9 (2)
C115—C116—H116	119.6	C215—C216—H216	119.6
C117—C116—H116	119.6	C217—C216—H216	119.6
C112—C117—C116	120.4 (2)	C216—C217—C212	120.05 (19)
C112—C117—H117	119.8	C216—C217—H217	120.0
C116—C117—H117	119.8	C212—C217—H217	120.0
			
C18—S11—C11—C12	−0.58 (16)	C28—S21—C21—C22	−0.23 (15)
C18—S11—C11—C19	178.95 (16)	C28—S21—C21—C29	−177.63 (16)
C19—C11—C12—C13	−178.81 (18)	C29—C21—C22—C23	177.56 (18)
S11—C11—C12—C13	0.7 (2)	S21—C21—C22—C23	0.4 (2)
C11—C12—C13—C14	178.41 (19)	C21—C22—C23—C24	179.47 (19)
C11—C12—C13—C18	−0.4 (2)	C21—C22—C23—C28	−0.5 (2)
C18—C13—C14—C15	0.8 (3)	C28—C23—C24—C25	0.1 (3)
C12—C13—C14—C15	−177.90 (19)	C22—C23—C24—C25	−179.82 (19)
C13—C14—C15—C16	−0.6 (3)	C23—C24—C25—C26	−0.1 (3)
C14—C15—C16—C17	−0.2 (3)	C24—C25—C26—C27	−0.2 (3)
C15—C16—C17—C18	0.8 (3)	C25—C26—C27—C28	0.5 (3)
C16—C17—C18—C13	−0.6 (3)	C26—C27—C28—C23	−0.5 (3)
C16—C17—C18—S11	177.95 (15)	C26—C27—C28—S21	179.33 (15)
C14—C13—C18—C17	−0.2 (3)	C24—C23—C28—C27	0.2 (3)
C12—C13—C18—C17	178.72 (17)	C22—C23—C28—C27	−179.86 (17)
C14—C13—C18—S11	−178.98 (14)	C24—C23—C28—S21	−179.65 (15)
C12—C13—C18—S11	0.0 (2)	C22—C23—C28—S21	0.3 (2)
C11—S11—C18—C17	−178.33 (19)	C21—S21—C28—C27	−179.87 (19)
C11—S11—C18—C13	0.33 (15)	C21—S21—C28—C23	−0.05 (15)
C12—C11—C19—N11	166.45 (19)	C22—C21—C29—N21	173.78 (18)
S11—C11—C19—N11	−13.0 (2)	S21—C21—C29—N21	−9.3 (2)
C12—C11—C19—C110	−13.9 (3)	C22—C21—C29—C210	−7.3 (3)
S11—C11—C19—C110	166.64 (15)	S21—C21—C29—C210	169.70 (15)
C11—C19—N11—N12	177.05 (16)	C21—C29—N21—N22	176.63 (16)
C110—C19—N11—N12	−2.6 (3)	C210—C29—N21—N22	−2.3 (3)
C19—N11—N12—C111	168.46 (17)	C29—N21—N22—C211	171.23 (18)
N11—N12—C111—O11	−177.39 (17)	N21—N22—C211—O21	176.31 (18)
N11—N12—C111—N13	1.1 (3)	N21—N22—C211—N23	−4.0 (3)
O11—C111—N13—C112	8.0 (3)	O21—C211—N23—C212	9.9 (3)
N12—C111—N13—C112	−170.44 (18)	N22—C211—N23—C212	−169.85 (18)
C111—N13—C112—C113	−13.3 (3)	C211—N23—C212—C217	−177.5 (2)
C111—N13—C112—C117	166.3 (2)	C211—N23—C212—C213	5.4 (3)
C117—C112—C113—C114	−1.6 (3)	C217—C212—C213—C214	−2.5 (3)
N13—C112—C113—C114	177.9 (2)	N23—C212—C213—C214	174.54 (19)
C112—C113—C114—C115	1.4 (4)	C212—C213—C214—C215	1.3 (3)
C113—C114—C115—C116	−0.3 (4)	C213—C214—C215—C216	0.3 (3)
C114—C115—C116—C117	−0.4 (3)	C214—C215—C216—C217	−0.6 (3)
C113—C112—C117—C116	0.9 (3)	C215—C216—C217—C212	−0.7 (3)
N13—C112—C117—C116	−178.7 (2)	C213—C212—C217—C216	2.2 (3)
C115—C116—C117—C112	0.2 (3)	N23—C212—C217—C216	−175.0 (2)

### Hydrogen-bond geometry (Å, °)

**Table:**

*Cg*1 is the centroid of the S11, C11, C12, C13, C18 ring.

**Table:**

*D*—H···*A*	*D*—H	H···*A*	*D*···*A*	*D*—H···*A*
N12—H12N···O21	0.97 (2)	1.91 (2)	2.847 (2)	162.1 (19)
N22—H22N···O11	0.89 (2)	1.99 (2)	2.840 (2)	158 (2)
C113—H113···O11	0.95	2.29	2.886 (2)	120.
C213—H213···O21	0.95	2.26	2.871 (2)	121.
C15—H15···O11^i^	0.95	2.62	3.435 (2)	144.
C24—H24···Cg1^ii^	0.95	2.80	3.482 (2)	130

Symmetry codes: (i) *x*, *y*+1, *z*; (ii) −*x*+1, −*y*+1, −*z*+1.

## References

[bb1] Alam, O., Mullick, P., Verma, S. P., Gilani, S. J., Khan, S. A., Siddiqui, N. & Ahsan, W. (2010). *Eur. J. Med. Chem.* **45**, 2467–2472. 10.1016/j.ejmech.2010.02.031 20211511

[bb2] Allen, F. H., Johnson, O., Shields, G. P., Smith, B. R. & Towler, M. (2004). *J. Appl. Cryst.* **37**, 335–338.

[bb3] Beraldo, H., Nacif, W. F. & West, D. X. (2001). *Spectrochim. Acta A*, **57**, 1847–1854. 10.1016/s1386-1425(01)00413-9 11506036

[bb4] Bernstein, J., Davis, R. E., Shimoni, L. & Chang, N.-L. (1995). *Angew. Chem. Int. Ed. Engl.* **34**, 1555–1573.

[bb5] Bruker (2009). *APEX2*, *SAINT* and *SADABS* Bruker AXS Inc., Madison, Wisconsin, USA.

[bb6] Fun, H.-K., Balasubramani, K., Vijesh, A. M., Malladii, S. & Isloor, A. M. (2009*a*). *Acta Cryst.* E**65**, o2072. 10.1107/S1600536809029900 PMC2969861 21577494

[bb7] Fun, H.-K., Yeap, C. S., Padaki, M., Malladi, S. & Isloor, A. M. (2009*b*). *Acta Cryst.* E**65**, o1807–o1808. 10.1107/S1600536809025847 PMC2977181 21583511

[bb8] Hunter, K. A. & Simpson, J. (1999). *TITAN2000* University of Otago, New Zealand.

[bb9] Macrae, C. F., Bruno, I. J., Chisholm, J. A., Edgington, P. R., McCabe, P., Pidcock, E., Rodriguez-Monge, L., Taylor, R., van de Streek, J. & Wood, P. A. (2008). *J. Appl. Cryst.* **41**, 466–470.

[bb10] Mendoza-Meroño, R., Menéndez-Taboada, L., Fernández-Zapico, E. & García-Granda, S. (2011). *Acta Cryst.* E**67**, o1135. 10.1107/S1600536811013134 PMC3089140 21754444

[bb11] Sharma, R., Agarwal, S. K., Rawat, S. & Nagar, M. (2006). *Transition Met* *Chem.* **31**, 201–206.

[bb12] Sheldrick, G. M. (2008). *Acta Cryst.* A**64**, 112–122. 10.1107/S0108767307043930 18156677

[bb13] Siji, V. L., Kumar, M. R. S., Suma, S. & Kurup, M. R. P. (2010). *Spectrochim. Acta A*, **76**, 22–28. 10.1016/j.saa.2010.02.035 20347382

[bb14] Spek, A. L. (2009). *Acta Cryst.* D**65**, 148–155. 10.1107/S090744490804362X PMC2631630 19171970

[bb15] Sriram, D., Yogeeswari, P. & Thirumurugan, R. (2004). *Bioorg. Med. Chem. Lett.* **14**, 3923–3924. 10.1016/j.bmcl.2004.05.060 15225698

[bb16] Westrip, S. P. (2010). *J. Appl. Cryst.* **43**, 920–925.

